# Root resorption after leveling with super-elastic and conventional steel arch wires: a prospective study

**DOI:** 10.1186/s40510-014-0035-z

**Published:** 2014-05-15

**Authors:** Kawa Alzahawi, Espen Færøvig, Pongsri Brudvik, Olav Egil Bøe, Maria Mavragani

**Affiliations:** Department of Clinical Dentistry-Orthodontics, Faculty of Medicine and Dentistry, University of Bergen, Årstadveien 19/21, Bergen, 5009 Norway; Department of Orthodontics, Dental Faculty, University of Oslo, Oslo, 0316 Norway; Department of Clinical Dentistry, Faculty of Medicine and Dentistry, University of Bergen, Bergen, 5009 Norway; Department of Community Dentistry, Section of Orthodontics, School of Dentistry, University of Athens, Athens, 10679 Greece

## Abstract

**Background:**

The aim of this prospective study was to compare root resorption after the leveling phase of treatment, performed by either super-elastic or conventional multi-stranded stainless steel arch wires.

**Methods:**

From a total of 156 future orthodontic patients in a private clinic, 82 were included in the study after excluding those who earlier had orthodontic or endodontic treatment or signs of resorption. Patients were equally arbitrary allocated into two groups, where leveling was performed either with super-elastic heat-activated or conventional multi-stranded stainless steel arch wires. Root length loss was calculated using pre-treatment and post-leveling periapical radiographs.

**Results:**

The use of super-elastic arch wires did not significantly increase the severity of root resorption, except for tooth 31, while it reduced leveling time compared to conventional stainless steel wires. Crossbite of maxillary lateral incisors seemed to be a risk factor for resorption.

**Conclusion:**

Incisor root resorption after leveling did not differ significantly between patients treated with super-elastic and conventional stainless steel arch wires, except for a mandibular incisor.

**Electronic supplementary material:**

The online version of this article (doi:10.1186/s40510-014-0035-z) contains supplementary material, which is available to authorized users.

## Background

Leveling and alignment are usually the first phase of orthodontic treatment with fixed appliances. It could be considered as the ‘trial’ phase, when patient's cooperation and tissue responses become apparent.

Among the biological reactions to tooth movement, tendency for apical root resorption during this early phase has been shown to be indicative of the final root length loss during the entire treatment [1, 2]. Even though mild apical root resorption is a usual finding among orthodontic patients [3], severe root length loss is quite infrequent. Levander and Malmgren [1] described resorption exceeding half the original root length in 1% of the tested teeth, while Linge and Linge [4] reported greater than 4 mm loss of root length in 2.3% of the treated teeth. Radiographs of the maxillary front teeth taken after 6 months of treatment can help to identify those patients at risk of severe apical root resorption [2].

Mechanotherapy during leveling phase of treatment is performed using arch wires that fall into two broad categories: stainless steel and nickel titanium with super-elastic properties. Stainless steel wires deliver a rapidly declining force, while super-elastic wires deliver a more constant force during the deactivation range [5]. Due to the convenience of the clinical use, super-elastic arch wires rapidly gained popularity among orthodontists, particularly during the leveling phase. A number of benefits for using super-elastic arch wires have been advocated; however, it has been reported that manufacturers were in such a rush to produce the ultimate aligning arch wire that very little attention has been paid to the *in vivo* behavior of these materials [6]. The systematic review by Riley and Bearn [7] on clinical trials on aligning arch wires advocated that there is insufficient data to make clear recommendations regarding the most effective arch wire for alignment. In a later Cohrane review by Wang et al. [8], some additional factors, including the amount of root resorption along with tooth movement and the intensity of pain experienced by patients during initial alignment, were evaluated. The authors concluded that there is some evidence to suggest that there is no difference between the speed of tooth alignment or pain experienced by patients when using one initial aligning arch wire over another. However, as they report, root resorption had not been investigated by any randomized clinical trial, even though it is one of the most serious side effects of orthodontic treatment. They suggest that further evaluation of the aligning arch wires should consider this potentially serious side effect of orthodontic treatment.

As derived from the literature, there seems to be lack of knowledge concerning the effect of the leveling arch wires used in everyday clinical practice on orthodontic root resorption. Therefore, the primary objective of this study was to compare the amount of root resorption following the leveling phase of orthodontic treatment performed with super-elastic arch wires and conventional multi-stranded stainless steel arch wires. Leveling duration was compared between the groups. Additionally, a comparison of the severity of apical root resorption between central and lateral incisors during that phase of treatment was attempted. Finally, the effect of several patient- and treatment-related variables on apical root resorption was evaluated.

## Methods

The study was approved by both the Ethical Committee in Norway and the Norwegian Social Science Data Services (NSD) (REK Vest 229.08 2008/12408-ØYSV). All study participants and/or their parents read and signed an informed consent form before treatment initiation. A power analysis was performed for calculation of the sample size. In order to identify a difference of 0.5 mm at the 0.05 level of significance with 80% power, using a standard deviation (SD) of 0.8, 41 patients were required in each group.

One hundred fifty-six orthodontic patients, who were about to start treatment at the private clinic of one of the authors (EF), were assessed for eligibility to participate in the study. Patients who showed signs of apical root resorption, were earlier orthodontically treated, or had endodontic treatment of their front teeth were excluded from the sample. Finally, 82 patients were included in this prospective study.

All patients received orthodontic treatment with straight-wire edgewise technique. The appliances used were 0.022-in. Diamond Twin brackets (Ormco Corp., Clendora, CA, USA). The study was parallel in design, with an allocation ratio of 1:1. Clinical assistants arbitrarily allocated patients into two experimental groups with 41 patients each, using prepared random number list (Table 1). Treatment was performed by one of the authors (EF), who was informed about the treatment group during bonding. During the leveling phase, patients in the first group were treated with super-elastic arch wires (SE group), whereas patients in the second group were treated with stainless steel arch wires (SS group). The typical arch wire sequence for the SE group was 0.014 in. Sentalloy, 0.016 in. Sentalloy, and 0.018 × 0.025 in. Bioforce (Dentsply/GAC, Bohemia, NY, USA). The arch wire sequence for the SS group was 0.0175 in. Penta-one multi-stranded (Masel Orthodontics, Carlsbad, CA, USA), 0.016 in. Australian regular (A J Wilcock PTY, LTD, Whittlesea, Australia), and 0.016 × 0.022 in. resilient (3M Unitek, Monrovia, CA, USA).

**Table 1:** **Gender and age distribution of the patients in the two groups**

	Males	Females	All	Mean age (years)	Range (years)	SD
SE group	22	19	41	13.3	11 to 16	1.5
SS group	18	23	41	13.7	11 to 22	2.4
All	40	42	82	13.5	11 to 22	2.0

A number of variables were recorded for each patient (gender, age, ANB angle, overjet, overbite, impacted maxillary canines, invagination of each incisor, crossbite separately for teeth 12 and 22). During the course of treatment, any complaint from the patients would be noted in their clinical journal. All patients were radiographically examined at two predetermined points of treatment: before treatment start and at the end of the leveling phase, approximately 6 to 10 months after initiation of treatment. Leveling duration was also recorded. During the examinations, periapical radiographs of maxillary and mandibular incisor teeth were taken with a standardized long-cone paralleling technique. The tube was placed at a fixed distance of 7 cm from the film, using a customized Eggen holder. Four radiographs were taken each time: one with the central ray beam between the two maxillary central incisors, one with the X-ray beam centered at the maxillary lateral incisor on each side, and one radiograph with the central beam between the mandibular central incisors. Both apex and the incisal edge of the incisor teeth should be clearly visible. Unsatisfactory X-rays were excluded from the study. Totally, 311 teeth were examined radiographically in 79 patients: 2 teeth in 6 patients, 3 in 18 patients, 4 in 46 patients, 5 in 1 patient, 6 teeth in 1 patient, 7 teeth in 6 patients, and finally 8 teeth in 1 patient. All periapical radiographs were converted to digital images by using an EPSON perfectionV700 scanner at a resolution of 300 dpi (Epson Inc., Long Beach, CA, USA). The Windows-based CliniView software package (Instrumentarium Dental, Tuusula, Finland) was used for adjustment in brightness and contrast between scanned images, and for measurements.

### Analysis of radiographs

**Figure 1:**
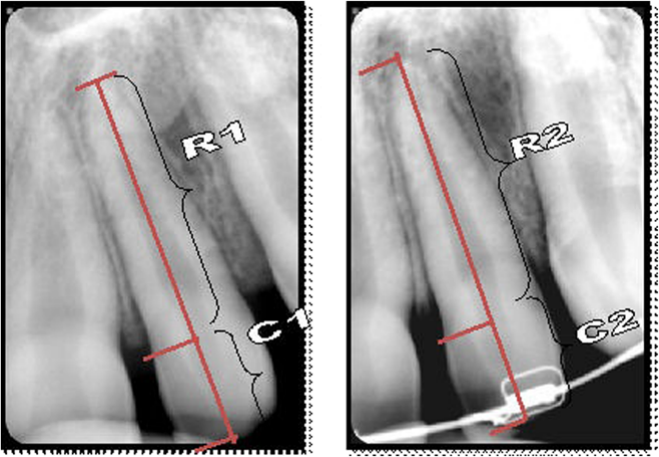
**Measurements on pre-treatment and post-leveling radiograms.** C1, crown length on pre-treatment radiogram, C2, crown length on post-leveling radiogram; R1, root length before treatment; R2, root length after leveling phase.

Root resorption has been estimated as any reduction in the radiographic length of all incisors from the cementoenamel junction (CEJ) to the root apex, after adjustment for different magnifications. The anatomic landmarks were demarcated on the X-ray films. For CEJ, the most distinct landmark either mesial or distal was used, but once selected, the same side was used for all radiograms of the tooth. The longitudinal axis of each tooth was constructed following the root canal. The marked CEJ, root apex, and incisal edge were projected perpendicular to this axis. Root length was measured from the projected CEJ to the apex on the constructed longitudinal axis, and crown length similarly to the incisal edge (Figure 1). A correction factor was calculated in order to relate the pre-treatment and post-leveling radiographs to each other. Root length was adjusted by using the crown length registrations, assuming crown length to be unchanged over the observation period. This method has earlier been described [9]. Its accuracy has been found satisfactory [10].

### Measurement error

All measurements were performed by one examiner (KA), who was blinded regarding the origin of the radiographs. The reproducibility of the measurements was assessed by analyzing the difference between double measurements by the same examiner on radiographs taken at least 2 weeks apart on 4 teeth (totally 72 teeth), one tooth on each side, both in the maxilla and mandible in 18 randomly selected patients. The systematic error between the double measurements was evaluated separately for each root length using the paired *t* test, and the measurement error on a single measurement was estimated by using the formula *τ* = SD/√2, where SD is the standard deviation of the differences between duplicated measurements.

### Statistical methods

In order to investigate whether the recorded variables, including leveling duration, differed significantly between the two treatment groups, a two-sample *t* test was carried out for the quantitative variables and a chi-square test for the qualitative variables. A two-sample *t* test was also used to test for any difference in root length reduction in millimeters and percentage between the two groups, for each examined tooth (12, 11, 21, 22, 32, 31, 41, and 42) separately.

To test for differences in root resorption between the central and lateral incisors, a paired *t* test was performed on the values of the central and the lateral incisors of the same quadrant of each patient. Finally, in order to assess any association between the percentage root shortening of each tooth and the other variables recorded, multiple linear regression analysis was applied. The statistical analyses were carried out using the SPSS software package (SPSS Inc., IBM Company, Chicago, IL, USA).

## Results

**Figure 2:**
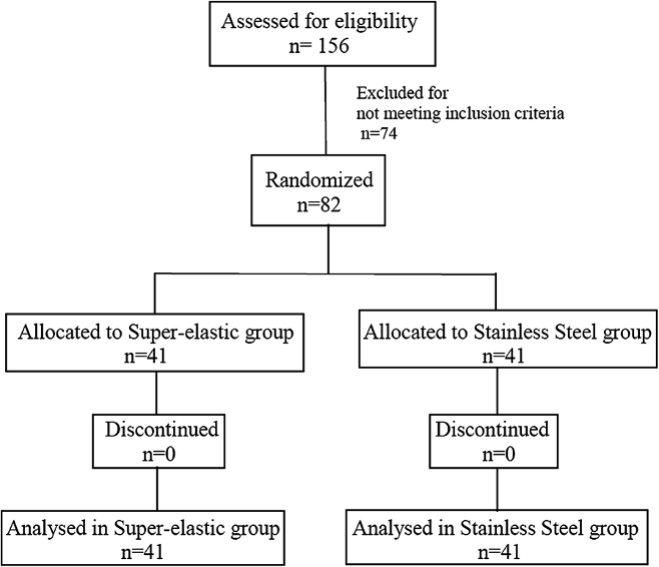
**CONSORT flowchart diagram of the clinical trial.**

None of the 41 allocated patients in each group was either lost to follow-up or discontinued. Finally, 41 patients in each group were analyzed (Figure 2). Patients' recruitment was performed during fall 2009, and the follow-up lasted until spring/summer 2010.

The two patient groups were well matched for all treatment variables except for leveling duration which was longer (*P* = 0.002; 95% CI = −1.31 to −0.30) for patients treated with stainless steel arch wires (mean 6.98 months, SD 1.42) than for patients treated with super-elastic arch wires (mean 6.17 months, SD 0.77), and invagination. Chi-square test revealed more invaginated incisors at the left side for the group treated with stainless steel than the one treated with super-elastic arch wires (tooth 21 (invaginated teeth out of a total of 41): super-elastic group 2, stainless steel group 11, *P* = 0.005; tooth 22 (invaginated teeth out of a total of 41): super-elastic group 4, stainless steel group 11, *P* = 0.04). No significant differences were revealed between groups for agenesis, trauma, and crossbites. Similarly, ANB angle (super-elastic: mean 2.8°, SD 2.15; stainless steel: mean 3.45°, SD 1.98; *P* = 0.85), overjet (super-elastic: mean 4.73 mm, SD 2.90; stainless steel: mean 4.68 mm, SD 2.77; *P* = 0.91), and overbite (super-elastic: mean 3.63 mm, SD 0.34; stainless steel: mean 3.61, SD 0.38; *P* = 0.52) did not differ between the two groups.

Statistical analysis of the difference in root resorption between the two treatment groups, in millimeters and in percentage of root shortening, for each tooth, revealed no significant results, except for tooth 31 that showed more resorption in the group treated with super-elastic arch wires (Table 2). Negative values, indicating root lengthening, were noted in most of the tooth groups. There was a tendency for more root resorption for the lateral than the central incisors both in the maxilla and mandible, with significant difference only between teeth 41 and 42 (Table 3).

**Table 2:** **Root resorption in millimeters and in percentage of the initial root length in the two groups**

	Super-elastic group				Stainless steel group				95% CI	***P***value
	***N***	Mean	SD	Range	***N***	Mean	SD	Range		
Tooth 12	19				19					
mm		1.07	0.91	0.00 to 2.90		0.91	1.09	−0.50 to 3.80	−0.50 to 0.82	0.62
%		6.69	5.78	0.00 to 18.24		5.14	5.42	−3.21 to 17.27	−2.14 to 5.24	0.40
Tooth 11	24				21					
mm		0.89	1.11	−1.20 to 3.60		0.68	0.68	−0.40 to 2.50	−0.36 to 0.77	0.46
%		5.33	6.81	−7.89 to 3.18		3.88	3.69	−2.38 to 12.69	−1.91 to 4.82	0.39
Tooth 21	23				21					
mm		0.53	0.92	−1.40 to 2.70		0.71	0.92	−0.90 to 2.90	−0.74 to 0.38	0.52
%		3.22	5.73	−8.38 to 8.49		3.88	5.36	−7.03 to 17.16	−4.05 to 2.72	0.70
Tooth 22	19				21					
mm		0.96	0.77	−0.10 to 2.50		0.65	0.52	0.00 to 1.80	−0.11 to 0.73	0.14
%		5.94	4.90	−0.55 to 6.67		3.96	3.11	0.00 to 12.16	−0.62 to 4.58	0.13
Tooth 32	18				13					
mm		0.94	0.77	0.00 to 2.90		1.14	0.88	0.00 to 3.40	−0.81 to 0.41	0.51
%		5.66	4.61	0.00 to 17.47		6.56	5.04	0.00 to 19.54	−4.48 to 2.66	0.61
Tooth 31	22				22					
mm		1.00	0.71	0.00 to 2.70		0.47	0.58	−0.30 to 1.90	0.13 to 0.92	0.01
%		6.32	4.15	0.00 to 15.88		2.95	3.61	−2.01 to 11.38	1.00 to 5.73	0.01
Tooth 41	23				21					
mm		0.97	1.09	0.00 to 4.00		0.61	0.46	0.00 to 1.60	−0.16 to 0.87	0.17
%		6.03	6.63	0.00 to 25.97		3.87	2.95	0.00 to 9.94	−1.02 to 5.33	0.18
Tooth 42	18				18					
mm		1.20	0.89	0.00 to 3.90		1.14	0.58	0.30 to 2.30	−0.46 to 0.57	0.83
%		6.88	4.86	0.00 to 19.60		6.41	3.09	1.67 to 12.71	−2.29 to 3.23	0.73

**Table 3:** **Root resorption in millimeters and percentage of initial root length for lateral and central incisors in maxilla and mandible**

	Mean	SD	Mean	SD	95% CI	***P***	Mean	SD	Mean	SD	95% CI	***P***
Pairs	12		11				22		21			
	(*N* = 37)						(*N* = 37)					
mm	0.97	1.00	0.74	0.87	−0.13 to 0.60	0.20	0.71	0.57	0.62	0.94	−0.23 to 0.42	0.55
%	5.79	5.60	4.30	5.02	−0.54 to 3.52	0.15	4.36	3.45	3.42	5.57	−1.04 to 2.92	0.34
Pairs	42		41				32		31			
	(*N* = 36)						(*N* = 30)					
mm	1.17	0.74	0.73	0.72	0.21 to 0.68	<0.01	1.04	0.82	0.79	0.65	−0.08 to 0.58	0.14
%	6.65	4.02	4.54	4.22	0.75 to 3.47	<0.01	6.14	4.78	5.07	4.02	−0.88 to 3.01	0.27

The results of the regression analysis, with the percentage of root shortening of the upper lateral incisors as dependent variable and all other variables as independent variables, revealed that apical root resorption of maxillary lateral incisors was significantly associated with the presence of crossbite of these teeth (Table 4). No other significant association between apical root resorption after leveling and other variables examined was shown for any tooth.

**Table 4:** **Regression analysis for teeth 12 and 22**

	Tooth 12				Tooth 22			
	Constant	Crossbite 12	Age	***R*** ^2^(adjusted) %	Constant	Crossbite 22	ANB	***R*** ^2^(adjusted) %
1	0.783	**0.967**		13.9	0.675	**0.600**		11.3
		*0.012*				*0.019*		
2	−1.149	**1.052**	**0.143**	17.6	0.975	**0.492**	**−0.091**	16.8
		*0.006*	*0.116*			*0.052*	*0.060*	
95% CI		0.32 to 1.79	−0.04 to 0.32			0.00 to 0.99	−0.19 to 0.01	

The figures in the first column denote the number of explanatory variables included in the regression analysis by first applying the best subset regression analysis among all variables. For each regression analysis, the partial regression coefficient of the variable is given in bold and the *P* value of the partial regression coefficient in italics.

No complaints for harm or unintended effects from patients under treatment were reported.

## Discussion

Only a limited number of teeth from each incisor planned to be examined were finally analyzed due to inappropriate radiographs. That may have a negative effect on the power of the study, since statistical analysis was performed for each tooth separately. This drawback could have been eliminated by providing additional training to the dental assistants for the specific requirements of the particular X-rays, mainly related to the full appearance of the incisal edge of the teeth.

The two groups did not differ considering the characteristics of malocclusion recorded, except for invagination for the left incisor teeth. Mild type of invagination, however, has been shown not to be a risk factor for orthodontic root resorption [11]. The severity of crowding has not been considered at this study since crowding determines to a large extent the amount of tooth movement during leveling, which could have influenced the results.

Negative values for root resorption indicating root length increase have also previously been reported and attributed either to a real increase in root length [4, 12] or to method error [13]. Error in registration of the CEJ, which is difficult to be identified, should also be considered.

The present study was performed in a sample of Norwegian orthodontic patients who were treated with straight-wire edgewise technique and conventional brackets. It could be assumed that the results from this study could be applicable for any patient treated with the same technique.

The end of leveling phase has been chosen as the time point for the evaluation of the effect of the two different arch wire materials, due to the possibility to use one type of arch wire material only, before the continuation of treatment that would require both types of arch wires. Additionally, the amount of root shortening 6 to 12 months after bracket placement is of high predictive value for the severity of root resorption after the completion of treatment [2]. Incisors were selected for this study because they are the most prone to tooth resorption, while a small reduction of their length is easier to be detected by conventional radiography. Most of the patients included in this study (90% of the examined teeth) exhibited signs of root resorption after initial treatment of 6 to 10 months. For the majority of the cases, the amount of resorption was minor, but present. According to Makedonas et al. [14], root resorption diagnosed by cone beam computed tomography after 6 months of treatment was clinically significant only for the 4% of the patients.

Leveling was accomplished sooner by super-elastic than stainless steel arch wires in our sample. Similarly, Weiland [15] has shown greater amount of buccal premolar displacement during the same period with super-elastic than stainless steel wires. Initial force applied by stainless steel wires soon declines and needs to be re-established, while the force induced by means of super-elastic wires continues over a longer time. Tooth movement with continuous forces was also found more effective than with interrupted continuous forces at the premolar tipping model by Owman-Moll et al. [16]. Longer treatment time with steel wires could be attributed to the intervals when steel wires are passive. However, in the Cochrane review of published randomized controlled trials (RCTs) by Wang et al. [8], no difference in the speed of tooth alignment could be shown after using one initial aligning arch wire over another. Authors commented on the general poor quality of the included trials and suggested that the results should be viewed with caution.

At the same Cohrane review, the lack of RCTs on the effect of different types of aligning arch wires on the severity of the induced root resorption was emphasized. At the experimental premolar buccal tipping by Weiland [15], root resorptive activity was found lower for the teeth moved by steel than super-elastic wires. The present study evaluated root resorption during the first phase of orthodontic treatment. Any type of tooth movement necessary for alignment was performed. No statistically significant difference was found between super-elastic and multi-stranded steel arch wires, except for the lower left central incisor which seemed to be significantly more affected by root resorption in the group treated with super-elastic arch wires. A general tendency for more severe apical root resorption in the super-elastic group was noticed, but that was only significant for tooth 31. Treatment duration that has been considered a risk factor for orthodontically induced root resorption [17–19] was longer for the stainless steel group. It could be hypothesized that the inactive intervals at force application, which may prolong treatment time, allow for repair of resorption lacunae. When orthodontic force is discontinued or falls under a certain level, lacunae are repaired by a process that is similar to the early cementogenesis occurring during tooth development [20, 21]. In other experimental animal studies, allowing for inactive periods of force application was beneficial against root resorption [22–24].

No significant difference in root resorption between the central and lateral incisors was revealed, except for the lower right incisors. There was a tendency, however, for more root resorption for the lateral incisors compared with the central incisors. Recent studies by Nanekrungsan et al. [25] and Lund et al. [3] found lateral incisors to be more often and seriously affected by root resorption.

An interesting finding from this study was the significant relation between crossbite and root resorption of the maxillary lateral incisors. That was not in agreement with Kaley and Phillips [26] who did not find any significant relation between crossbite and apical root resorption. In that study, the evaluation was performed at the end of orthodontic treatment, after the additive to the presently studied initial resorption effect of the subsequent phases of treatment. However, the authors recognized root torquing as risk factor for orthodontic resorption. The relation between crossbite and root resorption found in this study could be attributed to the increased amount of load at the apical thin area, when the tipping movement for the correction of the crossbite occurs. Teeth may be more prone to apical resorption during tipping than bodily movement, where pressure is more or less evenly distributed on the buccal and the lingual surfaces of the root [27]. It could also be possible that lateral incisors may had been subjected to traumatizing occlusal forces during the correction of the anterior crossbite, since the bite has not been raised to facilitate the correction of the crossbite in any of the cases. Even though Newman [28] did not find any relationship between root resorption and occlusal trauma or heavy occlusal forces, one could keep in mind that these factors can create jiggling movements. The latter has been proven to be a considerable risk factor for orthodontic root resorption [29].

## Conclusion

Root resorption of maxillary and mandibular incisors after leveling did not statistically differ among patients treated with super-elastic and those being treated with conventional stainless steel arch wires, except for the mandibular left central incisor that showed more extensive resorption in the super-elastic group. Crossbite seemed to be a risk factor for root resorption of the maxillary lateral incisors, during the initial stages of orthodontic treatment.

## Authors’ original submitted files for images

Below are the links to the authors’ original submitted files for images. Authors’ original file for figure 1 Authors’ original file for figure 2
