# Supplementary figures and images for: Root resorption after leveling with super-elastic and conventional steel arch wires: a prospective study
Source: Prog Orthod. 2014 May 15;15:35. doi: 10.1186/s40510-014-0035-z (PMC4883983; doi:10.1186/s40510-014-0035-z)

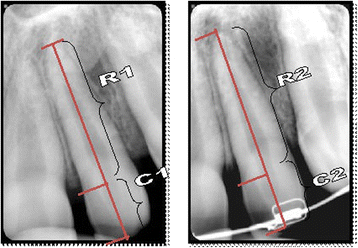

Supplement: Supplementary file 1 — Authors’ original file for figure 1 [file 40510_2014_35_MOESM1_ESM.gif]

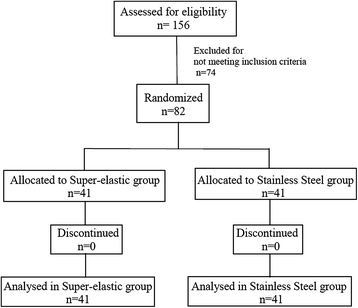

Supplement: Supplementary file 2 — Authors’ original file for figure 2 [file 40510_2014_35_MOESM2_ESM.gif]
